# Population-wide evaluation of artificial intelligence and radiologist assessment of screening mammograms

**DOI:** 10.1007/s00330-023-10423-7

**Published:** 2023-11-08

**Authors:** Johanne Kühl, Mohammad Talal Elhakim, Sarah Wordenskjold Stougaard, Benjamin Schnack Brandt Rasmussen, Mads Nielsen, Oke Gerke, Lisbet Brønsro Larsen, Ole Graumann

**Affiliations:** 1 Department of Clinical Research, University of Southern Denmark, Kløvervænget 10, 2ndfloor, 5000 Odense C, Denmark; 2 Department of Radiology, Odense University Hospital, Kløvervænget 47, Ground Floor, 5000 Odense C, Denmark; 3 CAI-X – Centre for Clinical Artificial Intelligence, Odense University Hospital, Kløvervænget 8C, 5000 Odense C, Denmark; 4 Department of Computer Science, University of Copenhagen, Universitetsparken 1, 2100 Copenhagen, Denmark; 5 Department of Nuclear Medicine, Odense University Hospital, Kløvervænget 47, 5000 Odense C, Denmark; 6 Department of Radiology, Aarhus University Hospital, Palle Juul-Jensens Blvd. 99, 8200 Aarhus N, Denmark; 7 Department of Clinical Research, Aarhus University, Palle Juul-Jensens Blvd. 99, 8200 Aarhus N, Denmark

**Keywords:** Mammography, Breast cancer, Artificial intelligence, Screening

## Abstract

**Objectives:**

To validate an AI system for standalone breast cancer detection on an entire screening population in comparison to first-reading breast radiologists.

**Materials and methods:**

All mammography screenings performed between August 4, 2014, and August 15, 2018, in the Region of Southern Denmark with follow-up within 24 months were eligible. Screenings were assessed as normal or abnormal by breast radiologists through double reading with arbitration. For an AI decision of normal or abnormal, two AI-score cut-off points were applied by matching at mean sensitivity (AI_sens_) and specificity (AI_spec_) of first readers. Accuracy measures were sensitivity, specificity, positive predictive value (PPV), negative predictive value (NPV), and recall rate (RR).

**Results:**

The sample included 249,402 screenings (149,495 women) and 2033 breast cancers (72.6% screen-detected cancers, 27.4% interval cancers). AI_sens_ had lower specificity (97.5% vs 97.7%; *p* < 0.0001) and PPV (17.5% vs 18.7%; *p* = 0.01) and a higher RR (3.0% vs 2.8%; *p* < 0.0001) than first readers. AI_spec_ was comparable to first readers in terms of all accuracy measures. Both AI_sens_ and AI_spec_ detected significantly fewer screen-detected cancers (1166 (AI_sens_), 1156 (AI_spec_) vs 1252; *p* < 0.0001) but found more interval cancers compared to first readers (126 (AI_sens_), 117 (AI_spec_) vs 39; *p* < 0.0001) with varying types of cancers detected across multiple subgroups.

**Conclusion:**

Standalone AI can detect breast cancer at an accuracy level equivalent to the standard of first readers when the AI threshold point was matched at first reader specificity. However, AI and first readers detected a different composition of cancers.

**Clinical relevance statement:**

Replacing first readers with AI with an appropriate cut-off score could be feasible. AI-detected cancers not detected by radiologists suggest a potential increase in the number of cancers detected if AI is implemented to support double reading within screening, although the clinicopathological characteristics of detected cancers would not change significantly.

**Key Points:**

• *Standalone AI cancer detection was compared to first readers in a double-read mammography screening population.*

• *Standalone AI matched at first reader specificity showed no statistically significant difference in overall accuracy but detected different cancers.*

• *With an appropriate threshold, AI-integrated screening can increase the number of detected cancers with similar clinicopathological characteristics.*

**Supplementary Information:**

The online version contains supplementary material available at 10.1007/s00330-023-10423-7.

## Introduction

Breast cancer is the leading cause of cancer-related deaths amongst women [1]. Systematic screening has been proven efficient in detecting breast cancer at early, less advanced stages and reducing overall breast cancer-specific mortality [2, 3]. The quality of screening varies with the quantity of resources, mammographic image characteristics, and the accuracy and experience level of readers [4–6]. For organized screening of women aged 50–69 years, the European Commission Initiative on Breast Cancer recommends double reading of mammograms with consensus or arbitration for discordant readings [7]. Such screening programs require a large capacity of specialized radiologists in a field highly affected by staff shortages [8, 9].

Integration of artificial intelligence (AI) solutions in breast cancer screening has shown potential to help overcome capacity issues, standardize accuracy, and improve efficiency [10–12]. Possible implementation sites range from the application as a reader-aid to functioning as a standalone reader for triage or replacement of radiologists [11, 13]. One study investigated different scenarios with AI as a standalone reader and found it theoretically possible to reduce screen reading volume without reducing cancer detection rates [14]. While a recent systematic review on standalone AI breast cancer detection found that the time has come to investigate implementation strategies [12], other reviews have considered the existing evidence insufficient to recommend implementation in real-world settings [10, 11, 15]. The European Commission Initiative on Breast Cancer currently recommends the implementation of AI only as a reader aid for support in double reading with arbitration or consensus reading [16]. Limitations in current literature include cancer-enriched or small datasets, low generalizability, and non-representative reference standards. Hence, there is a lack of consecutive cohorts representative of a screening population with a reliable reference standard [10, 15].

This study aimed to validate a deep learning-based AI system for standalone breast cancer detection on a consecutive cohort of mammograms representative of an entire screening population with a setting of double reading and arbitration. Specifically, the objectives were to (i) determine the standalone detection accuracy of the AI system, and (ii) compare the accuracy of the AI system to that of first reading breast radiologists.

## Materials and methods

### Study design

Ethics approval was granted by the Danish National Committee on Health Research Ethics (identifier D1763009). The study followed the Standard for Reporting of Diagnostic Accuracy Studies (STARD) reporting guideline (Supplementary eMethod 1) [17]. This was a multicenter accuracy study performed on a retrospective cohort of digital mammograms from an entire regional screening population.

### Study population

The Region of Southern Denmark offers biennial mammography screening to asymptomatic women aged 50–69 years. Women with a history of breast cancer can participate until the age of 79, while women with genetic predisposition are offered lifelong screening. All mammograms performed in the screening program in the Region of Southern Denmark between August 4, 2014, and August 15, 2018, were eligible for inclusion. The study period was selected to ensure the inclusion of two consecutive national screening rounds and a sufficient follow-up period. Regional breast cancer centers were in the cities of Odense, Vejle, Esbjerg, and Aabenraa that cover 1.22 million inhabitants of which approximately 75,000 women constitute an entire target population for screening within the region. The examinations were excluded in case of missing images, lack of follow-up, insufficient image quality, or image data types not supported by the AI system.

### Data collection

A consecutive image dataset was extracted in raw DICOM format from local radiology archives by using the women’s unique Danish identification number. Mammograms were performed on a single mammography vendor (Siemens Mammomat Inspiration, Siemens Healthcare A/S). Images included a minimum of one craniocaudal and/or mediolateral oblique projection of at least one breast per screening. Screening data including assessment results and information on the reviewing radiologists were extracted from the joint regional Radiological Information System. All mammograms were originally assessed through blinded double reading with a binary decision outcome as either normal or abnormal. Arbitration, i.e., a third reading, was performed in case of discordant readings. The arbitrator had access to the decisions of both the first and second readers. Abnormal outcomes resulted in a recall for a diagnostic work-up at a dedicated breast imaging unit. Clearly defined criteria for the designation of radiologists into first and second reader positions in Denmark do not exist. However, in practice, second readers tend to have more experience than first readers. The position of the arbitrator is routinely allotted to the most experienced radiologists, which, though, could have screen-read the same mammogram. Data on the experience level of radiologists were self-reported, with “Years engaged in reading screening mammograms” as the variable of interest. Follow-up information on breast cancer diagnosis and tumor characteristics was obtained by matching with the database of the Danish Breast Cancer Cooperative Group (DBCG) and the Danish Quality Database on Mammography Screening (DKMS) [18, 19], obtained via the Danish Clinical Quality Program – National Clinical Registries (RKKP).

### Artificial intelligence system

All mammograms were analyzed by the commercially available AI system Lunit INSIGTH MMG (v.1.1.7.1, Lunit Inc.), CE-marked and FDA-approved for concurrent reading aid. The AI system is based on a deep learning model that provides a per-view abnormality score of 0–100%, for which a score of 100% signifies the highest suspicion of malignancy. The maximum of the per-view scores was used to define an exam-level Lunit score for the study, which was further dichotomized into an AI score to enable comparability with the binary reader outcomes. Two different thresholds were explored, AI_sens_ and AI_spec_, which were matched at mean sensitivity and specificity of first reader outcome, respectively, with outcomes above the threshold considered as recalls. These thresholds were chosen to enable testing and comparison of the AI system at a level equivalent to a well-defined group of radiologists in terms of breast cancer detection. The choice of these two thresholds would also ensure approximately equivalent numbers of false positive recalls or missed cancers, respectively, should AI replace the first reader in a real-life AI-integrated screening. The AI system did not include clinical data, previous mammograms, or screening results in the assessment. The mammograms in this study have never been used for training, validation, or testing of the AI system.

### Reference standard

Positive cancer outcomes were determined by a documented breast cancer diagnosis, including non-invasive breast cancer, i.e., ductal carcinoma in situ, following recall from screening (screen-detected cancer) or before the next consecutive screening within 24 months (interval cancer). Negative cancer outcomes were defined by cancer-free follow-up up until next screening or within 24 months. Follow-up data on cancer outcomes was extracted from the DKMS and DBCG registries.

### Statistical analysis

Binomial proportions for the accuracy of AI and radiologists were calculated and supplemented by 95% Clopper-Pearson (“exact”) confidence intervals (CI). McNemar’s test or exact binomial test, when discordant cells were too small, was used to compare the accuracy of AI and radiologists, while t-test was used to evaluate associations for continuous variables. Measures of accuracy were sensitivity and specificity as co-primary outcomes, and positive predictive value (PPV), negative predictive value (NPV), and recall rate (RR) as secondary outcomes. The analysis was supplemented with empirical receiver operating characteristic (ROC) curves with corresponding area under the curve (AUC) values, for which asymptotic normal CIs were applied. The co-primary and secondary outcomes were determined for all radiologists by reading position (first reader, second reader, arbitrator, and combined reading) and for the standalone AI system for each of the two thresholds (AI_sens_ and AI_spec_). The outcome of the arbitrator was calculated as that of the other readers but was based on a selected group of screen-read disagreements between the first reader and second reader, making it a smaller number of screenings.

Specific subgroup analyses compared the detection rates across age and cancer subgroups, including histological subtype, tumor size, malignancy grade, TNM stage, lymph node positivity, estrogen receptor status, and HER2 status. To explore and compare the ability of AI and first readers in early breast cancer detection, an exploratory analysis of cancer detection accuracy was carried out while including next-round screen-detected cancers (diagnosed at next consecutive screening) and long-term cancers (diagnosed > 2–7 years after screening) in the positive cancer outcomes. For this purpose, linear regression with the measure of performance as outcome was used to take the correlation between women and possible multiple cancers into account. *p* < 0.05 was considered statistically significant. All statistical analyses were carried out using Stata/SE (Release 17, www.stata.com).

## Results

### Study sample

**Fig. 1:**
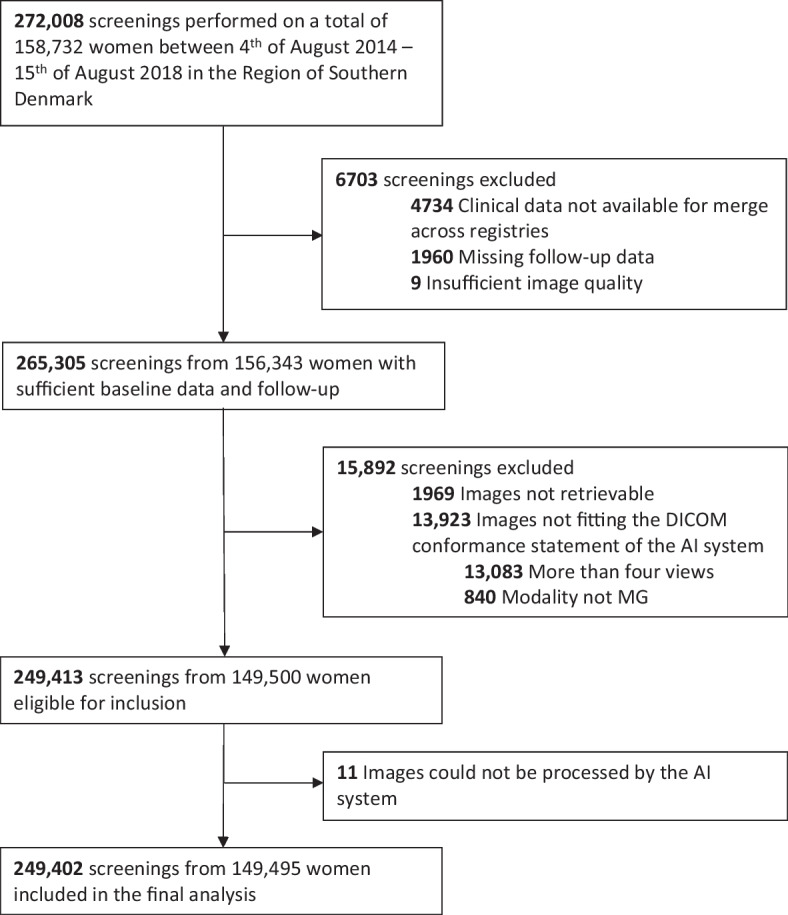
Study flowchart. The study included screening examinations from an entire mammography screening population across two screening rounds in the Region of Southern Denmark. Due to the biennial screening interval, multiple consecutive screening examinations from a single woman could be included. Abbreviation: AI, artificial intelligence. MG, mammography

A total of 272,008 screenings were performed within the inclusion period. Of these, 22,606 mammograms (8.3%) were excluded from the analyses (Fig. 1). Thus, 249,402 mammograms from 149,495 women were included in the study sample (Table 1). The sensitivity analysis showed a statistically significant difference across variables for excluded screenings with sufficient data and follow-up (*n* = 15,892), although absolute differences were found small (Supplementary eTable 1).

**Table 1:** Screening examination characteristics

Characteristic	Study sample (*n* = 249,402)
Screening site	
Odense	101,260 (40.6)
Vejle	52,253 (21.0)
Esbjerg	48,120 (19.3)
Aabenraa	47,769 (19.2)
Age in years at screening, mean [SD]	59.3 [5.9]
< 50^a^	25 (< 0.1)
50–59	130,332 (52.3)
60–69	116,390 (46.7)
70–79	2601 (1.0)
≥ 80	54 (< 0.1)
Breast cancer	2033 (0.8)
Screen-detected cancer	1475 (72.6)
Interval cancer	558 (27.4)
Breast cancer type	
Invasive cancer	1826 (89.8)
DCIS	207 (10.2)
Screening outcome	
Normal	242,555 (97.3)
Abnormal (recall)	6847 (2.8)
Agreement between readers	
First and second reader	242,395 (97.2)
First reader and arbitrator	3294 (44.3)
Second reader and arbitrator	4553 (61.2)
Reader position by experience in years	
First reader	249,402 (100.0)
0–4	140,972 (56.5)
5–9	78,755 (31.6)
10 +	29,601 (11.9)
Unknown	74 (< 0.1)
Second reader	249,402 (100.0)
0–4	79,323 (31.8)
5–9	382 (0.2)
10 +	169,697 (68.0)
Arbitrator^b^	7438 (3.0)
0–4	664 (8.9)
5–9	15 (0.2)
10 +	6759 (90.9)

Data are presented as n (%) of examinations unless otherwise indicated

Abbreviation: *DCIS*, ductal carcinoma in situ

^a^ Women in this age group were all 49 years old and received their first regular screening a few months early

^b^ Number of arbitrations are equivalent to the arbitration rate in the study sample. A small overlap of *n* = 431 (0.2%) examinations is observed in the number of arbitrations and agreements between first and second readers due to disagreements on subset outcomes that were ultimately classified into the available binary screening outcome

The included number of breast cancers was 2033 (0.8%), which included 1475 (72.6%) screen-detected cancers and 558 (27.4%) interval cancers. A total of 23 radiologists were involved in the screen reading. All 23 figured as first readers, 14 as second readers, and 10 were arbitrators. Screenings read by first readers were, in most cases (56.5%), by radiologists with 0–4 years of experience, while 68.0% of second readings and 90.9% of arbitrations were by radiologists with 10 + years of experience. The first and second readers agreed upon the screening outcome in 97.2% of cases.

### Radiologist detection accuracy

Accuracy outcomes for the AI system and all readers are presented in Table 2, with ROC curves and AUC values reported in Supplementary eFigure 1. The first reader had a sensitivity of 63.5% (95% CI 61.4–65.6%) and a specificity of 97.7% (97.7–97.8%). The second reader and combined reading achieved higher sensitivity and specificity than the first reader (*p* < 0.0001 for all). The arbitrator had a higher sensitivity with a markedly lower specificity (*p* < 0.0001 for both), although this was anticipated considering that only flagged examinations reached arbitration. A comparison between the screening outcome and the results of the reference standard is further detailed in Supplementary eTable 2.

**Table 2:** Cancer detection accuracy of the artificial intelligence system and the radiologists across reader position

	First reader	Second reader	Arbitrator	Combined reading	Standalone AI_sens_^a^	Standalone AI_spec_^b^
Sensitivity^c^	63.5 (61.4–65.6); ref	67.8 (65.8–69.9); < 0.0001	91.2 (88.0–93.7); < 0.0001	74.0 (72.1–75.9); < 0.0001	63.6 (61.4–65.6); > 0.99	62.6 (60.5–64.7); 0.43
Specificity^c^	97.7 (97.7–97.8); ref	98.0 (97.9–98.0); < 0.0001	52.5 (51.4–53.7); < 0.0001	97.8 (97.8–97.9); < 0.0001	97.5 (97.5–97.6); < 0.0001	97.7 (97.7–97.8); 0.96
PPV^d^	18.7 (17.8–19.6); ref	21.7 (20.6–22.7); < 0.0001	10.0 (9.1–11.0); < 0.0001	22.0 (21.0–23.0); < 0.0001	17.5 (16.7–18.4); 0.01	18.4 (17.5–19.4); 0.64
NPV^d^	99.7 (99.7–99.7); ref	99.7 (99.7–99.8); 0.001	99.0 (98.7–99.3); < 0.0001	99.8 (99.8–99.8); < 0.0001	99.7 (99.7–99.7); 0.99	99.7 (99.7–99.7); 0.51
RR^d^	2.8 (2.7–2.8); ref	2.6 (2.5–2.6); < 0.0001	49.9 (48.7–51.0); < 0.0001	2.7 (2.7–2.8); 0.40	3.0 (2.9–3.0); < 0.0001	2.8 (2.7–2.8); 0.89

Data are % (95% CI); *p* value

Abbreviations: *AI*_*sens*_, artificial intelligence score cut-off point matched at mean sensitivity of the first reader outcome; *AI*_*spec*_, artificial intelligence score cut-off point matched at mean specificity of the first reader outcome; *PPV*, positive predictive value; *NPV*, negative predictive value; *RR*, recall rate

^a^ AI_sens_: a Lunit score of 79.75%

^b^ AI_spec_: a Lunit score of 80.99%

^c^ *p* values were calculated using McNemar’s test

^d^ *p* values were calculated using exact binomial test

### AI score benchmarking

**Fig. 2:**
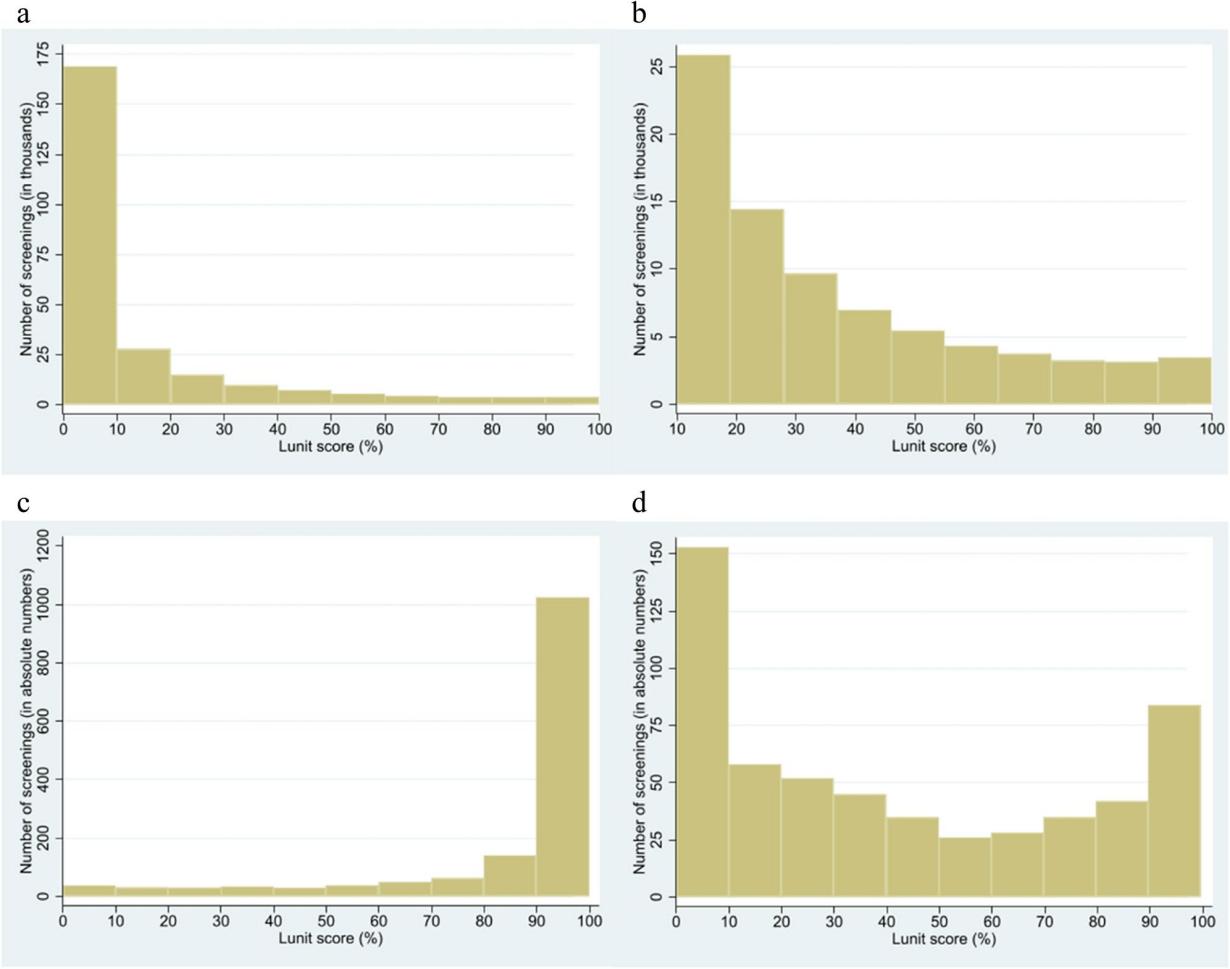
Distribution of abnormality scores across the study sample and all cancers. **a** The distribution of Lunit abnormality scores across all screening examinations in the study sample. A score of 100% signifies the highest suspicion of malignancy.** b** Enlargement of the score distribution across screening examinations given a score ≥ 10%.** c** The distribution of Lunit abnormality scores across screening examinations with a screen-detected cancer.** d** The score distribution across screening examinations from women diagnosed with an interval cancer

When matching by mean first reader sensitivity and specificity, the cut-off points for AI_sens_ and AI_spec_ were Lunit Score 79.75% and 80.99%, respectively. The distribution of Lunit scores across screenings is depicted in Fig. 2.

### Cancer detection accuracy of Standalone AI

Standalone AI_spec_ did not differ statistically significantly from the first reader across any accuracy measures (Table 2). Standalone AI_sens_ showed statistically significantly lower specificity (− 0.2%) and higher RR (+ 0.2%) than first reader (*p* < 0.0001 for both). The breakdown of accuracy by cancer subgroups, as presented in Table 3, showed fewer screen-detected cancers by Standalone AI_sens_ (− 5.8%) and Standalone AI_spec_ (− 6.5%) compared to the first reader (*p* < 0.0001 for both). However, Standalone AI_sens_ and Standalone AI_spec_ detected more interval cancers by + 15.6% and + 14.0%, respectively (*p* < 0.0001 for both). In terms of tumor characteristics, Standalone AI at both thresholds found more 21–50 mm cancers and more lymph node-positive cancers. Yet, when detection rates were stratified across screen-detected cancers and interval cancers, these findings only applied to the latter, while the opposite was the case for screen-detected cancers (Supplementary eTable 3). This pattern of lower and higher accuracy across screen-detected cancers and interval cancers, respectively, was observed for more than half of cancer subgroups, for which Standalone AI at both thresholds had statistically significantly different detection rates compared to the first reader. Moreover, subgroup analyses of detection agreements and discrepancies between the AI and first reader showed that Standalone AI at both thresholds disagreed with the first reader in 23% of all cancer cases, which were either detected by AI and missed by the first reader or vice versa (Supplementary eTable 4).

**Table 3:** Subgroup analysis of detection rates across cancer subgroups for all cancers

	Detected by		
	First reader	Standalone AI_sens_	Standalone AI_spec_
Age in years at screening (*n* = 2033)	60.9 [6.0]; ref	60.6 [6.0]; 0.18^a^	60.6 [6.0]; 0.20^a^
All cancers (*n* = 2033)	1291 (63.5); ref	1292 (63.6); > 0.99	1273 (62.6); 0.43
Screen-detected cancer (*n* = 1475)	1252 (84.9); ref	1166 (79.1); < 0.0001	1156 (78.4); < 0.0001
Interval cancer (*n* = 558)	39 (7.0); ref	126 (22.6); < 0.0001	117 (21.0); < 0.0001
< 12 months after screening (*n* = 175)	14 (8.0); ref	50 (28.6); < 0.0001	47 (26.9); < 0.0001
≥ 12 months after screening (*n* = 383)	25 (6.5); ref	76 (19.8); < 0.0001	70 (18.3); < 0.0001
Histological subtype			
Invasive ductal (*n* = 1387)	900 (64.9); ref	902 (65.0); 0.95	888 (64.9); 0.53
Invasive lobular (*n* = 228)	118 (51.8); ref	119 (52.2); > 0.99	119 (52.2); > 0.99
Other invasive (*n* = 211)	102 (48.3); ref	104 (49.3); 0.90	101 (47.9); > 0.99
DCIS (*n* = 207)	171 (82.6); ref	167 (80.7); 0.69	165 (79.7); 0.51
Tumor size^b^			
0–10 mm (*n* = 568)	394 (69.4); ref	350 (61.6); 0.001	347 (61.1); 0.0003
11–20 mm (*n* = 795)	522 (65.7); ref	524 (65.9); 0.93	514 (64.7); 0.56
21–50 mm (*n* = 377)	174 (46.2); ref	213 (56.5); < 0.0001	211 (56.0); < 0.0001
51 + mm (*n* = 47)	16 (34.0); ref	21 (44.7); 0.30	19 (40.4); 0.61
Unknown (*n* = 39)	14 (35.9); ref	17 (43.6); 0.51	17 (43.6); 0.51
Malignancy grade^b^			
Grade 1 (*n* = 518)	335 (64.7); ref	345 (66.6); 0.40	342 (66.0); 0.57
Grade 2 (*n* = 814)	518 (63.6); ref	523 (64.3); 0.77	515 (63.3); 0.88
Grade 3 (*n* = 349)	189 (54.2); ref	184 (52.7); 0.64	180 (51.6); 0.34
Unknown (*n* = 145)	78 (53.8); ref	73 (50.3); 0.52	71 (49.0); 0.32
TNM stage^b^			
Local (I + II) (*n* = 1755)	1100 (62.7); ref	1098 (62.6); 0.96	1081 (61.6); 0.36
Locally advanced (III) (*n* = 44)	16 (36.4); ref	16 (36.4); 1.00	16 (36.4); 1.00
Distant metastasis (IV) (*n* = 21)	4 (19.2); ref	9 (42.9); 0.06	9 (42.9); 0.06
Unknown (*n* = 6)	0 (0.0); ref	2 (33.3); < 0.001^c^	2 (33.3); < 0.001^c^
Lymph node positivity^b^			
No (*n* = 1329)	834 (62.8); ref	812 (61.1); 0.23	800 (60.2); 0.06
Yes (*n* = 497)	286 (57.6); ref	313 (63.0); 0.01	308 (62.0); 0.04
ER positivity^b^			
0% (*n* = 205)	94 (45.9); ref	85 (41.5); 0.21	84 (41.0); 0.16
1–9% (*n* = 107)	49 (45.8); ref	42 (39.3); 0.26	39 (36.5); 0.06
10–100% (*n* = 1502)	971 (64.7); ref	992 (66.1); 0.28	979 (65.2); 0.70
Unknown (*n* = 12)	6 (50.0); ref	6 (50.0); 1.00	6 (50.0); 1.00
HER2 status^b^			
Negative (*n* = 1579)	987 (62.5); ref	985 (62.4); 0.96	970 (61.4); 0.40
Positive (*n* = 226)	124 (54.9); ref	130 (57.5); 0.44	128 (56.6); 0.65
Unknown (*n* = 21)	9 (42.9); ref	10 (47.6); 1.00	10 (47.6); 1.00

All numbers are n (%); *p* value or mean [SD]; *p* value

Abbreviations: *AI*_*sens*_, artificial intelligence score cut-off point matched at mean sensitivity of the first reader outcome; *AI*_*spec*_, artificial intelligence score cut-off point matched at mean specificity of the first reader outcome; *DCIS*, ductal carcinoma in situ; *TNM*, tumor, node, and metastasis; *ER*, estrogen receptor; *HER2*, human epidermal growth factor receptor 2

^a^ *p* values were calculated using t-test

^b^ Reported for invasive cancers only

^c^ Exact binomial test used instead of McNemar’s test due to small discordant cells

Exploratory analysis of cancer detection when including next-round screen-detected and long-term cancers showed statistically significantly higher sensitivity of the AI system at both thresholds than first readers (*p* < 0.0001 for both), as presented in Table 4.

**Table 4:** Cancer detection accuracy analysis with inclusion of next-round screen-detected cancers and long-term cancers

	First reader	Standalone AI_sens_	Standalone AI_spec_
Sensitivity^a^	31.5 (30.1–32.8); ref	35.9 (34.5–37.4); < 0.0001	35.1 (33.7–36.6); < 0.0001
Specificity^a^	97.7 (97.7–97.8); ref	97.6 (97.6–97.7); 0.01	97.8 (97.8–97.9); 0.08
PPV^b^	20.2 (19.3–21.2); ref	21.7 (20.7–22.7); 0.002	22.6 (21.6–23.7); < 0.0001
NPV^b^	98.7 (98.7–98.8); ref	98.8 (98.8–98.9); 0.0004	98.8 (98.8–98.9); 0.003

Data are % (95% CI); *p* value. Next-round screen-detected cancers (*n* = 1166) and Long-term cancers (*n* = 2219) include ductal carcinoma in situ

Abbreviations: *AI*_*sens*_, artificial intelligence score cut-off point matched at mean sensitivity of the first reader outcome in the total study sample. *AI*_*spec*_, artificial intelligence score cut-off point matched at mean specificity of the first reader outcome in the total study sample; *PPV*, positive predictive value; *NPV*, negative predictive value

^a^ *p* values were calculated using McNemar’s test

^b^ *p* values were calculated using exact binomial test

## Discussion

### Main findings

We obtained a large representative study sample with a cancer detection rate and recall rate, which were in agreement with previously reported screening outcomes from Danish screening rounds [19, 20]. This study observed two main findings. Firsty, the cancer detection accuracy of Standalone AI_spec_ was not statistically significantly different from the first reader across any accuracy measure. Standalone AI_sens_, however, had a lower specificity and higher recall rate than first readers. Secondly, the AI system exhibited a statistically significantly lower detection rate of screen-detected cancers but a higher detection of interval cancers compared to the first reader at both AI thresholds and a higher accuracy when taking next-round screen-detected cancers and long-term cancers into account. This was expected in the context of a retrospective study design where the AI detection of screen-detected cancers was compared directly to the readers who detected the cancers. However, the AI system detected different cancers than the first reader, even for Standalone AI_spec_, which exhibited equivalent reading accuracy to the first reader. The observations of a generally lower and higher AI accuracy across screen-detected cancers and interval cancers, respectively, for more than half of the cancer subgroups (Supplementary eTable 3), along with detection discrepancies of a notable number of cancers in the agreement analysis (Supplementary eTable 4), suggest that AI in combination with human readers in double reading could result in an increase in the number of cancers detected. The differences in cancer detection accuracy should also be considered in relation to the clinical relevance of the cancers detected and their malignancy potential. Standalone AI at both thresholds was not equal to or better than first readers at detecting small cancers sized 0–10 mm or grade 3 tumors (Table 3), which both are indicators of high malignancy [21]. These findings suggest that the AI system is not necessarily more capable than first readers at detecting cancers reflecting tumor aggressive potential, which is an important consideration in proportion to the implementation of AI. Notwithstanding, the findings indicate that the clinicopathological characteristics of detected cancers would overall remain unaltered in an AI-supported screening setup.

### Comparison with current literature

Other studies investigating standalone AI have reported varying accuracy estimates [10]. The accuracy of standalone AI found in this study was consistent with results from Salim et al [22]. They assessed three independent AI systems on a selected, double-reader screening cohort and found the best-performing AI to exceed the sensitivity of first readers when tested at an AI score threshold matched at first reader specificity [22]. Both Rodriguez-Ruiz et al and McKinney et al found similar results with the accuracy of their standalone AI systems being non-inferior to and superior to single readers, respectively [23, 24]. Although similar findings are observed, these and other previously published validation studies [25, 26] show discrepancies in their methodological approaches regarding choice of AI score threshold and comparator, among others. For instance, Rodriguez-Ruiz et al matched the AI threshold at the average specificity of single readers across several international datasets, including both general and breast radiologists from the US and Europe, with varying quality assurance standards [24]. McKinney et al chose different thresholds in their UK training set, depending on which group of readers figured as comparator and then applied the algorithm with those thresholds to a separate US test set for comparison [23]. Their intent was to find a threshold where the accuracy of the AI exceeded or was non-inferior to the average reader. Moreover, most studies on standalone AI cancer detection have, in several systematic reviews, been assessed to suffer from a high risk of bias or low level of generalizability mostly due to cancer enrichment, selection biases, and/or varying reference standard [10, 11, 15]. More recently, a few studies with large population-based cohorts have been published that methodologically minimize some limitations. Lauritzen et al included an unselected Danish screening sample and found significantly lower specificity for standalone AI when the AI score threshold was matched at sensitivity, although this was in comparison to the consensus of both radiologists in double reading [27]. A large study by Leibig et al reported significantly lower sensitivity and specificity of standalone AI compared to a single radiologist when the threshold was set to maintain the radiologist’s sensitivity [28]. This study, however, excluded > 35% of participants and used adjusted sample weighting in the external test set to compensate for oversampling with cancer enrichment, introducing a high risk of selection bias.

### Strengths and limitations

In contrast, the major strength of our study was the large representative sample of unselected, consecutive mammograms from an entire screening population. High-quality data from multiple national registries were used to ensure a comprehensive sample and reliable reference standard. Although our findings might transfer to similar populations, accuracy estimates could differ in populations with a different breast cancer prevalence or significantly different population characteristics.

Among the limitations in our work is the lack of a gold standard for all screening mammograms, i.e., verification bias, as examinations with a positive AI outcome, not recalled by the radiologists, were not offered diagnostic work-up due to the retrospective nature of this study [29]. This potentially skews the estimation of the AI accuracy. The exploratory analysis, including next-round screen-detected and long-term cancers (Table 4) partially solves this issue and could potentially be a more accurate approximation of the actual accuracy outcome. Additional bias is introduced as the reference standard is correlated to the manual reads by radiologists. Women who were recalled after screening had a higher chance of being diagnosed with breast cancer than those marked as normal at screening, potentially skewing the detection accuracy in favor of the human readers. Another limitation is that around 5% of screening examinations were excluded due to technical issues or mammograms not fitting the DICOM conformance statement. It should be noted that some of the limitations of the AI system, such as the inability to process screening exams with more than 4 views, are, according to the developers, mainly related to the experimental usage of retrospective data and not necessarily present in an AI-integrated screening on site. This and similar technical limitations are important for decision-makers to consider when planning the implementation of an AI solution in clinical practice. However, for this study, these exclusions are estimated not to have had any significant impact on the findings, seeing as the study sample was found to be representative of the study population. A final limitation is that the thresholds for the AI system were derived from the study dataset. Using a prespecified threshold would have been a more objective and generalizable approach than setting the sensitivity or specificity of the readers from the same dataset as benchmark. Although this was not possible due to the lack of a binary outcome intrinsic to the AI system, our approach is still valid from a clinical applicability point of view as the minimal anticipated cancer detection accuracy of the AI is no lower than the current standard. To the best of our knowledge, no specific methodological approach has been recommended in this matter. Most previous studies have chosen the same approach as this study or solely matched on either sensitivity or specificity [22, 24–28, 30]. As exemplified by Larsen et al, researchers should consider testing AI systems at several thresholds depending on the intended site of integration, to ensure reliable and realistic estimates of the accuracy of the AI before actual implementation [14].

### Perspectives and implications

Our paper, along with other studies, contribute to accurate estimates of breast cancer detection on screening mammograms by different AI systems, which can serve as a breeding ground for the design of future research and recommendations on sites for AI integration [31]. A survey study on attitudes towards the implementation of AI in breast cancer screening showed that UK readers are mostly supportive of partial replacement with AI replacing one reader [32]. The Danish Health Authorities recently recommended implementing AI as a replacement for first readers if investigations show supportive findings [33]. In view of the findings and considerations made in this study, AI accuracy estimates should ideally match the accuracy of the given group of readers that the AI is intended to replace. In addition, one issue that needs to be considered prior to clinical deployment is how the removal of radiologists, for instance, from the first reader position, might affect the detection accuracy of the other reader groups or the screening workflow in general. The accuracy of radiologists is, among other things, associated with the individual annual reading volume [5]. While there is no official standard in terms of designating radiologists into first and second reader positions in Denmark, the most experienced radiologists are ordinarily allotted to the second reader position. This tendency was confirmed in this study, as the majority of first readings were by radiologists with 0–4 years of experience, and most second readers had 10 + years of experience. Replacing first reader with an AI system could present the dilemma of how radiologists without the experience gained through first reading will achieve high levels of accuracy to sustain the high standard achieved through double reading. Implementing AI for triaging mammograms of low suspicion to single reading might lead to similar issues, as high-volume exposure to normal images is important to improve the discriminatory ability of radiologists [5]. These and other issues relating to potential implications in screening practice could be further elucidated in rigorously designed studies on AI-integrated screening, most optimally in the form of prospective randomized controlled trials, which should focus on how integration affects workload and final screening outcome and finding an optimal site of application in a long-term perspective. The first of such studies have most recently emerged, showing promising results from real-world clinical practice of AI-integrated population-based screening [34, 35].

### Conclusions

The accuracy of the AI system was comparable to that of first readers in a double-read mammography screening population, mainly when the AI score cut-off was matched at first reader specificity, highlighting the importance of choosing an appropriate threshold. The AI system and first readers detected different cancers, suggesting that integration of AI in a double reading setting could increase the number of cancers detected at screening without markedly changing the clinicopathological characteristics.

### Supplementary Information

Below is the link to the electronic supplementary material.

Supplementary file1 (DOCX 319 KB)

## Abbreviations

AI: Artificial intelligence
AI_sens_: Artificial intelligence score cut-off point matched at mean sensitivity of the first reader outcome
AI_spec_: Artificial intelligence score cut-off point matched at mean specificity of the first reader outcome
AUC: Area under the curve
CE: Conformité Européenne (European Conformity)
CI: Confidence interval
DBCG: Danish Breast Cancer Cooperative Group
DCIS: Ductal carcinoma in situ
DICOM: Digital Imaging and Communications in Medicine
DKMS: Danish Quality Database on Mammography Screening
ER: Estrogen receptor
FDA: Food and Drug Administration
HER2: Human epidermal growth factor receptor 2
MG: Mammography
NVP: Negative predictive value
PPV: Positive predictive value
RKKP: Danish Clinical Quality Program – National Clinical Registries
ROC: Receiver operating characteristic
RR: Recall rate
STARD: Standard for Reporting of Diagnostic Accuracy Studies
TNM: Tumor, nodes, and metastases

## Declarations

### Guarantor

The scientific guarantor of this publication is Benjamin Schnack Brandt Rasmussen.

### Statistics and biometry

One of the authors has significant statistical expertise: Oke Gerke.

No complex statistical methods were necessary for this paper.

### Study subjects or cohorts overlap

Some study subjects or cohorts have been previously reported in another related study by our group and are currently under consideration for publication elsewhere.

That study validates a different AI solution based on the same image and registry data. Hence, the index test, study sample, and subsequently, all results differ from the current study. Furthermore, the methodology in terms of the testing of the index test also differs.

### Methodology

• retrospective

• diagnostic or prognostic study

• multicenter study
