# Supplementary material for: Population-wide evaluation of artificial intelligence and radiologist assessment of screening mammograms
Source: Eur Radiol. 2023 Nov 8;34(6):3935–46. doi: 10.1007/s00330-023-10423-7 (PMC11166831; doi:10.1007/s00330-023-10423-7)
Supplement: Supplementary file 1 — Supplementary file1 (DOCX 319 KB) [file 330_2023_10423_MOESM1_ESM.docx]

**SUPPLEMENTARY MATERIAL**

**Population-wide evaluation of artificial intelligence and radiologist assessment of screening mammograms**

**Contents**

1. Supplementary eMethod 1: Standard for Reporting of Diagnostic Accuracy Studies (STARD) checklist p 2
2. Supplementary eFigure 1: Receiver operating characteristic (ROC) curves with area under the curve (AUC) values p 3
3. Supplementary eTable 1: Drop-out analysis p 4
4. Supplementary eTable 2: Comparison of screening outcome and results of the reference standard p 5
5. Supplementary eTable 3: Detection rates across cancer subgroups for screen-detected cancers and interval cancers p 6
6. Supplementary eTable 4: Subgroup analysis of cancer detection agreements and discrepancies across cancer subgroups p 7

**Supplementary eMethod 1: Standard for Reporting of Diagnostic Accuracy Studies (STARD) checklist**

|  | **Section & Topic** | **No** | **Item** | **Reported on page # (submitted version)** |
| --- | --- | --- | --- | --- |
|  |  |  |  |  |
|  | **TITLE OR ABSTRACT** |  |  |  |
|  |  | **1** | Identification as a study of diagnostic accuracy using at least one measure of accuracy (such as sensitivity, specificity, predictive values, or AUC) | 1 |
|  | **ABSTRACT** |  |  |  |
|  |  | **2** | Structured summary of study design, methods, results, and conclusions  (for specific guidance, see STARD for Abstracts) | 1 |
|  | **INTRODUCTION** |  |  |  |
|  |  | **3** | Scientific and clinical background, including the intended use and clinical role of the index test | 3 |
|  |  | **4** | Study objectives and hypotheses | 3-4 |
|  | **METHODS** |  |  |  |
|  | *Study design* | **5** | Whether data collection was planned before the index test and reference standard were performed (prospective study) or after (retrospective study) | 4 |
|  | *Participants* | **6** | Eligibility criteria | 4 |
|  |  | **7** | On what basis potentially eligible participants were identified  (such as symptoms, results from previous tests, inclusion in registry) | 4-5 |
|  |  | **8** | Where and when potentially eligible participants were identified (setting, location and dates) | 4-5 |
|  |  | **9** | Whether participants formed a consecutive, random or convenience series | 4-5 |
|  | *Test methods* | **10a** | Index test, in sufficient detail to allow replication | 5-8 |
|  |  | **10b** | Reference standard, in sufficient detail to allow replication | 6 |
|  |  | **11** | Rationale for choosing the reference standard (if alternatives exist) | - |
|  |  | **12a** | Definition of and rationale for test positivity cut-offs or result categories  of the index test, distinguishing pre-specified from exploratory | 6-7 |
|  |  | **12b** | Definition of and rationale for test positivity cut-offs or result categories  of the reference standard, distinguishing pre-specified from exploratory | 6-7 |
|  |  | **13a** | Whether clinical information and reference standard results were available  to the performers/readers of the index test | 5-6 |
|  |  | **13b** | Whether clinical information and index test results were available  to the assessors of the reference standard | 6 |
|  | *Analysis* | **14** | Methods for estimating or comparing measures of diagnostic accuracy | 6-7 |
|  |  | **15** | How indeterminate index test or reference standard results were handled | 4 |
|  |  | **16** | How missing data on the index test and reference standard were handled | 4 |
|  |  | **17** | Any analyses of variability in diagnostic accuracy, distinguishing pre-specified from exploratory | 6-7 |
|  |  | **18** | Intended sample size and how it was determined | - |
|  | **RESULTS** |  |  |  |
|  | *Participants* | **19** | Flow of participants, using a diagram | Figure 1 |
|  |  | **20** | Baseline demographic and clinical characteristics of participants | 8 + Table 1 |
|  |  | **21a** | Distribution of severity of disease in those with the target condition | 8 + Table 1 |
|  |  | **21b** | Distribution of alternative diagnoses in those without the target condition | - |
|  |  | **22** | Time interval and any clinical interventions between index test and reference standard | - |
|  | *Test results* | **23** | Cross tabulation of the index test results (or their distribution)  by the results of the reference standard | Table 2 + eTable 2 |
|  |  | **24** | Estimates of diagnostic accuracy and their precision (such as 95% confidence intervals) | 8-9 + Table 2-4 + eFigure 1 + eTable 3 |
|  |  | **25** | Any adverse events from performing the index test or the reference standard | - |
|  | **DISCUSSION** |  |  |  |
|  |  | **26** | Study limitations, including sources of potential bias, statistical uncertainty, and generalisability | 12-13 |
|  |  | **27** | Implications for practice, including the intended use and clinical role of the index test | 10-14 |
|  | **OTHER INFORMATION** |  |  |  |
|  |  | **28** | Registration number and name of registry | 4 |
|  |  | **29** | Where the full study protocol can be accessed | - |
|  |  | **30** | Sources of funding and other support; role of funders | See Funding section |
|  |  |  |  |  |
|  |  |  |  |  |

**Supplementary eFigure 1: Receiver operating characteristic (ROC) curves with area under the curve (AUC) values**

**
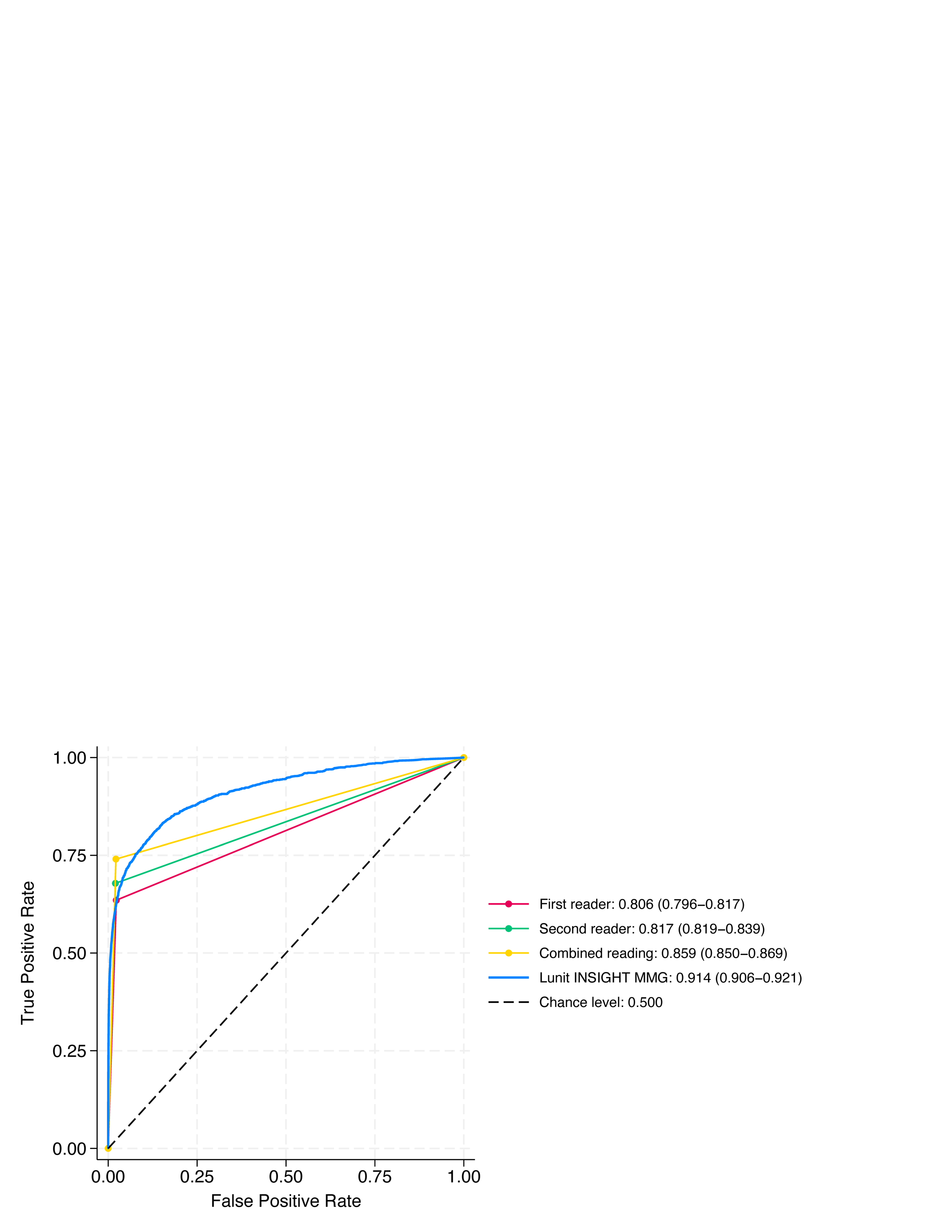
**

Empirical receiver operating characteristics curves showing the area under the curve for the outcome of first reader, second reader, combined reading, and the AI system over its entire operating range of exam level scores (Lunit score).

**Supplementary eTable 1: Drop-out analysis**

| Characteristic | Eligible screenings  (n = 249,413) | Exclusions (n = 15,892) | Odds ratio | 95% CI | *p* value |
| --- | --- | --- | --- | --- | --- |
| Screening site  Odense  Vejle  Esbjerg  Aabenraa | 101,267 (40.6)  52,254 (21.0)  48,123 (19.3)  47,769 (19.2) | 7014 (44.1)  2245 (14.1)  3457 (21.8)  3176 (20.0) | Ref.  0.64  1.07  0.96 | 0.60-0.67  1.02-1.11  0.92-1.01 | <0.001  0.003  0.087 |
| Age in years at screening  <50  50-59  60-69  70-79  ≥80 | 59.3 [5.9]  25 (<0.1)  130,335 (52.3)  116,398 (46.7)  2601 (1.0)  54 (<0.1) | 62.3 [7.1]  0 (0.0)  5849 (36.8)  7520 (47.3)  2469 (15.5)  54 (0.3) | 1.09 | 1.08-1.09 | <0.0001 |
| Breast cancer  Screen-detected cancer  Interval cancer | 2033 (0.8)  1475 (72.6)  558 (27.4) | 292 (1.8)  176 (60.3)  116 (39.7) | 1.27  Ref.  2.60 | 0.83-1.92  1.96-3.45 | 0.268  <0.0001 |
| Breast cancer type  Invasive cancer  DCIS | 1826 (89.8)  207 (10.2) | 266 (91.1)  26 (8.9) | 0.86 Ref. | 0.56-1.34 | 0.515 |
| Screening outcome  Normal  Abnormal (recall) | 242,566 (97.3)  6847 (2.8) | 15,287 (96.2)  605 (3.8) | Ref.  1.48 | 1.34-1.64 | <0.0001 |

Data are presented as n (%) or mean [SD].

Logistic regression with exclusions (yes/no) as outcome and the variables: screening site, age in years at screening, breast cancer, interval cancer, invasive cancer, and screening outcome as predictors, with robust standard errors.

Abbreviations: CI, confidence interval; DCIS, ductal carcinoma in situ.

**Supplementary eTable 2: Comparison of screening outcome and results of the reference standard**

|  | First reader | Second reader | Combined reading | Standalone AI_sens_ | Standalone AI_spec_ |
| --- | --- | --- | --- | --- | --- |
| True positives | 1291 (ref.) | 1379 (+6.6) | 1505 (+15.3) | 1292 (+0.1) | 1273 (-1.4) |
| True negatives | 241,743 (ref.) | 242,380 (+0.3) | 242,027 (+0.1) | 241,294 (-0.2) | 241,737 (-<0.1) |
| False positives | 5626 (ref.) | 4989 (-12.0) | 5342 (-5.2) | 6075 (+7.7) | 5632 (+0.1) |
| False negatives | 742 (ref.) | 654 (-12.6) | 528 (-33.7) | 741 (-0.1) | 760 (+2.4) |
| Recalls | 6917 (ref.) | 6368 (-8.3) | 6847 (-1.0) | 7367 (+6.3) | 6905 (-0.2) |

Data are n (Δ%). Abbreviations: AI_sens_, artificial intelligence score cut-off point matched at mean sensitivity of the first reader outcome; AI_spec_, artificial intelligence score cut-off point matched at mean specificity of the first reader outcome.

|  | Arbitrator |
| --- | --- |
| True positives | 371 |
| True negatives | 3694 |
| False positives | 3337 |
| False negatives | 36 |
| Recalls | 3708 |

Data are n. To avoid inconsistency, the numbers for the arbitrator are reported separately as the total number of screenings (n=7,438) differs from the other readers and the AI where n=249,402, and the differences can therefore not be compared to the reference in a similar manner.

**Supplementary eTable 3: Detection rates across cancer subgroups for screen-detected cancers and interval cancers**

|  | First reader | Standalone AI_sens_ | Standalone AI_spec_ |
| --- | --- | --- | --- |
| Screen-detected cancers (n=1268)^a^ |  | | |
| Tumor size  0-10 mm (n=484)  11-20 mm (n=576)  21-50 mm (n=179)  51+ mm (n=14)  Unknown (n=15) | 386 (79.8); ref.  510 (88.5); ref.  160 (89.4); ref.  11 (78.6); ref.  14 (93.3); ref. | 336 (69.4); <0.0001  478 (83.0); 0.002  161 (89.9); >0.99  13 (92.9); 0.33^b^  11 (73.3); 0.01^b^ | 334 (69.0); <0.0001  473 (82.1); 0.0004  161 (89.9); >0.99  12 (85.7); 0.75^b^  11 (73.3); 0.01^b^ |
| Malignancy grade  Grade 1 (n=409)  Grade 2 (n=575)  Grade 3 (n=188)  Unknown (n=96) | 327 (80.0); ref.  502 (87.3); ref.  176 (93.6); ref.  76 (79.2); ref. | 324 (79.2); 0.84  456 (79.3); <0.0001  155 (82.5); 0.001  64 (66.7); 0.04 | 323 (79.0); 0.76  452 (78.6); <0.0001  153 (81.4); 0.0004  63 (65.6); 0.02 |
| TNM stage  Local (I + II) (n=1252)  Locally advanced (III) (n=11)  Distant metastasis (IV) (n=5) | 1066 (85.1); ref.  11 (100.0); ref.  4 (80.0); ref. | 986 (78.8); <0.0001  9 (81.8); <0.0001^b^  4 (80.0); >0.99^b^ | 978 (78.1); <0.0001  9 (81.8); <0.0001^b^  4 (80.0); >0.99^b^ |
| Lymph node positivity  No (n=962)  Yes (n=306) | 809 (84.1); ref.  272 (88.9); ref. | 743 (77.2); <0.0001  256 (83.7); 0.04 | 736 (76.5); <0.0001  255 (83.3); 0.03 |
| ER positivity  0% (n=93)  1-9% (n=52)  10-100% (n=1116)  Unknown (n=7) | 87 (93.6); ref.  46 (88.5); ref.  942 (84.4); ref.  6 (85.7); ref. | 69 (74.2); 0.0003  31 (59.6); 0.0003  893 (80.0); 0.002  6 (85.7); >0.99^b^ | 68 (73.1); 0.0002  30 (57.7); 0.0001  887 (79.5); 0.001  6 (85.7); >0.99^b^ |
| HER2 status  Negative (n=1127)  Positive (n=130)  Unknown (n=11) | 954 (84.7); ref.  118 (90.8); ref.  9 (81.8); ref. | 882 (78.3); <0.0001  109 (83.9); 0.09  8 (72.7); >0.99 | 876 (77.7); <0.0001  107 (82.3); 0.04  8 (72.7); >0.99 |
| Interval cancers (n=558)^a^ |  | | |
| Tumor size  0-10 mm (n=84)  11-20 mm (n=219)  21-50 mm (n=198)  51+ mm (n=33)  Unknown (n=24) | 8 (9.5); ref.  12 (5.5); ref.  14 (7.1); ref.  5 (15.2); ref.  0 (0.0); ref. | 14 (16.7); 0.18  46 (21.0); <0.0001  52 (26.3); <0.0001  8 (24.2); 0.55  6 (25.0); <0.0001^b^ | 13 (15.5); 0.27  41 (18.7); <0.0001  50 (25.3); <0.0001  7 (21.2); 0.75  6 (25.0); <0.0001^b^ |
| Malignancy grade  Grade 1 (n=109)  Grade 2 (n=239)  Grade 3 (n=161)  Unknown (n=49) | 8 (7.3); ref.  16 (6.7); ref.  13 (8.1); ref.  2 (4.1); ref. | 21 (19.3); 0.01  67 (28.0); <0.0001  29 (18.0); 0.01  9 (18.49); 0.07 | 19 (17.4); 0.02  63 (26.4); <0.0001  27 (16.8); 0.02  8 (16.3); 0.11 |
| TNM stage  Local (I + II) (n=503)  Locally advanced (III) (n=33)  Distant metastasis (IV) (n=16)  Unknown (n=6) | 34 (6.8); ref.  5 (15.2); ref.  0 (0.0); ref.  0 (0.0); ref. | 112 (22.3); <0.0001  7 (21.2); 0.73  5 (31.3); <0.0001^b^  2 (33.3); <0.0001^b^ | 103 (20.5); <0.0001  7 (21.2); 0.73  5 (31.3); <0.0001^b^  2 (33.3); <0.0001^b^ |
| Lymph node positivity  No (n=376)  Yes (n=191) | 25 (6.8); ref.  14 (7.3); ref. | 69 (18.8); <0.0001  57 (29.8); <0.0001 | 64 (17.4); <0.0001  53 (27.8); <0.0001 |
| ER positivity  0% (n=112)  1-9% (n=55)  10-100% (n=386)  Unknown (n=5) | 7 (6.3); ref.  3 (5.5); ref.  29 (7.5); ref.  0 (0.0); ref. | 16 (14.3); 0.03  11 (20.0); 0.04  99 (25.7); <0.0001  0 (0.0); >0.99^b^ | 16 (14.3); 0.049  9 (16.4); 0.11  92 (23.8); <0.0001  0 (0.0); >0.99^b^ |
| HER2 status  Negative (n=452)  Positive (n=96)  Unknown (n=10) | 33 (7.3); ref.  6 (6.3); ref.  0 (0.0); ref. | 103 (22.8); <0.0001  21 (21.9); 0.001  2 (20.0); <0.0001^b^ | 94 (20.8); <0.0001  21 (21.9); 0.001  2 (20.0); <0.0001^b^ |

Data are n (%); *p* value. The cancer detection rate is reported out of the total in each subgroup.

Abbreviations: AI_sens_, artificial intelligence score cut-off point matched at mean sensitivity of the first reader outcome; AI_spec_, artificial intelligence score cut-off point matched at mean specificity of the first reader outcome; TNM, Tumor, Node and Metastasis; ER, estrogen receptor; HER2, Human Epidermal Growth Factor Receptor 2.

^a^ Reported for invasive cancers only.

^b^ Exact binomial test was used instead of McNemar’s test due to small discordant cells.

**Supplementary eTable 4: Subgroup analysis of cancer detection agreements and discrepancies across cancer subgroups**

|  | Detected by both first reader and Standalone: | | Detected by first reader,  missed by Standalone: | | Missed by first reader,  detected by Standalone: | | Missed by both first reader  and Standalone: | |
| --- | --- | --- | --- | --- | --- | --- | --- | --- |
|  | **AI_sens_** | **AI_spec_** | **AI_sens_** | **AI_spec_** | **AI_sens_** | **AI_spec_** | **AI_sens_** | **AI_spec_** |
| All cancers (n=2033) | 1057 (52.0) | 1049 (51.6) | 234 (11.5) | 242 (11.9) | 235 (11.6) | 224 (11.0) | 507 (24.9) | 518 (25.5) |
| Screen-detected cancer (n=1475) | 1038 (70.4) | 1030 (69.8) | 214 (14.5) | 222 (15.1) | 128 (8.7) | 126 (8.5) | 95 (6.4) | 97 (6.6) |
| Interval cancer (n=558)  <12 months after screening (n=175)  ≥12 months after screening (n=383) | 19 (3.4)  4 (2.3)  15 (3.9) | 19 (3.4)  4 (2.3)  15 (3.9) | 20 (3.6)  10 (5.7)  10 (2.6) | 20 (3.6)  10 (5.7)  10 (2.6) | 107 (19.2)  46 (26.3)  61 (15.9) | 98 (17.6)  43 (24.6)  55 (14.4) | 412 (73.8)  115 (65.7)  297 (77.5) | 421 (75.4)  118 (67.4)  303 (79.1) |
| Histological subtype  Invasive ductal (n=1387)  Invasive lobular (n=228)  Other invasive (n=211)  DCIS (n=207) | 751 (54.1)  92 (40.4)  74 (35.1)  140 (67.6) | 744 (53.6)  92 (40.4)  74 (35.1)  139 (67.1) | 149 (10.7)  26 (11.4)  28 (13.3)  31 (15.0) | 156 (11.2)  26 (11.4)  28 (13.3)  32 (15.5) | 151 (10.9)  27 (11.8)  30.0 (14.2)  27 (13.0) | 144 (10.4)  27 (11.8)  27 (12.8)  26 (12.6) | 336 (24.2)  83 (36.4)  79 (37.4)  9 (4.3) | 343 (24.7)  83 (36.4)  82 (38.9)  10 (4.8) |
| Tumor size^a^  0-10 mm (n=568)  11-20 mm (n=795)  21-50 mm (n=377)  51+ mm (n=47)  Unknown (n=39) | 292 (51.4)  40 (56.6)  153 (40.6)  11 (23.4)  11 (28.2) | 290 (51.1)  446 (56.1)  153 (40.6)  10 (21.3)  11 (28.2) | 102 (18.0)  72 (9.1)  21 (5.6)  5 (10.6)  3 (7.7) | 104 (18.3)  76 (9.6)  21 (5.6)  6 (12.8)  3 (7.7) | 58 (10.2)  74 (9.3)  60 (15.9)  10 (21.3)  6 (15.4) | 57 (10.0)  68 (8.6)  58 (15.4)  9 (19.1)  6 (15.4) | 116 (20.4)  199 (25.0)  143 (37.9)  21 (44.7)  19 (48.7) | 117 (20.6)  205 (25.8)  145 (38.5)  22 (46.8)  19 (48.7) |
| Malignancy grade^a^  Grade 1 (n=518)  Grade 2 (n=814)  Grade 3 (n=349)  Unknown (n=145) | 283 (54.6)  427 (52.5)  151 (43.3)  56 (38.6) | 282 (54.4)  423 (52.0)  149 (42.7)  56 (38.6) | 52 (10.0)  91 (11.2)  38 (10.9)  22 (15.2) | 53 (10.2)  95 (11.7)  40 (11.5)  22 (15.2) | 62 (12.0)  96 (11.8)  33 (9.5)  17 (11.7) | 60 (11.6)  92 (11.3)  31 (8.9)  15 (10.3) | 121 (23.4)  200 (24.6)  127 (36.4)  50 (34.5) | 123 (23.7)  204 (25.1)  129 (37.0)  52 (35.9) |
| TNM stage^a^  Local (I + II) (n=1755)  Locally advanced (III) (n=44)  Distant metastasis (IV) (n=21)  Unknown (n=6) | 902 (51.4)  11 (25.0)  4 (19.0)  0 (0.0) | 895 (51.0)  11 (25.0)  4 (19.0)  4 (19.0) | 198 (11.3)  5 (11.4)  0 (0.0)  0 (0.0) | 205 (11.7)  5 (11.4)  0 (0.0)  0 (0.0) | 196 (11.2)  5 (11.4)  5 (23.8)  2 (33.3) | 186 (10.6)  5 (11.4)  5 (23.8)  5 (23.8) | 459 (26.2)  23 (52.3)  12 (57.1)  4 (66.7) | 469 (26.7)  23 (52.3)  12 (57.1)  4 (66.7) |
| Lymph node positivity^a^  No (n=1329)  Yes (n=497) | 673 (50.6)  244 (49.1) | 667 (50.2)  243 (48.9) | 161 (12.1)  42 (8.5) | 167 (12.6)  43 (8.7) | 139 (10.5)  69 (13.9) | 133 (10.0)  65 (13.1) | 356 (26.8)  142 (28.6) | 362 (27.2)  146 (29.4) |
| ER positivity^a^  0% (n=205)  1-9% (n=107)  10-100% (n=1502)  Unknown (n=12) | 69 (33.7)  31 (29.0)  812 (54.1)  5 (41.7) | 68 (33.2)  30 (28.0)  807 (53.7)  5 (41.7) | 25 (12.2)  18 (16.8)  159 (10.6)  1 (8.3) | 26 (12.7)  19 (17.8)  164 (10.9)  1 (8.3) | 16 (7.8)  11 (10.3)  180 (12.0)  1 (8.3) | 16 (7.8)  9 (8.4)  172 (11.5)  1 (8.3) | 95 (46.3)  47 (43.9)  351 (23.4)  5 (41.7) | 95 (46.3)  49 (45.8)  359 (23.9)  5 (41.7) |
| HER2 status^a^  Negative (n=1579)  Positive (n=226)  Unknown (n=21) | 804 (50.9)  106 (46.9)  7 (33.3) | 799 (50.6)  104 (46.0)  7 (33.3) | 183 (11.6)  18 (8.0)  2 (9.5) | 188 (11.9)  20 (8.8)  2 (9.5) | 181 (11.5)  24 (10.6)  3 (14.3) | 171 (10.8)  24 (10.6)  3 (14.3) | 411 (26.0)  78 (34.5)  9 (42.9) | 421 (26.7)  78 (34.5)  9 (42.9) |

All numbers are n (%); *p* value.

Abbreviations: AI_sens_, artificial intelligence score cut-off point matched at mean sensitivity of the first reader outcome; AI_spec_, artificial intelligence score cut-off point matched at mean specificity of the first reader outcome; DCIS, Ductal carcinoma in situ; TNM, Tumor, Node and Metastasis; ER, estrogen receptor; HER2, Human Epidermal Growth Factor Receptor 2.

**^a^** Reported for invasive cancers only.
